# White Matter Microstructure Predicts Autistic Traits in Attention-Deficit/Hyperactivity Disorder

**DOI:** 10.1007/s10803-014-2131-9

**Published:** 2014-05-15

**Authors:** Miriam Cooper, Anita Thapar, Derek K. Jones

**Affiliations:** 1 Child and Adolescent Psychiatry Section, Institute of Psychological Medicine and Clinical Neurosciences, Cardiff University School of Medicine, Second Floor, Hadyn Ellis Building, Maindy Road, Cathays, Cardiff, CF24 4HQ UK; 2 MRC Centre for Neuropsychiatric Genetics and Genomics, Cardiff University School of Medicine, Cardiff, UK; 3 Cardiff University Brain Research Imaging Centre, Cardiff University School of Psychology, Cardiff, UK; 4 Neuroscience and Mental Health Research Institute, Cardiff University, Cardiff, UK

**Keywords:** Diffusion MRI, ADHD, ASD, White matter, Tract-based spatial statistics, RESTORE

## Abstract

Traits of autism spectrum disorder (ASD) in children with attention-deficit/hyperactivity disorder (ADHD) have previously been found to index clinical severity. This study examined the association of ASD traits with diffusion parameters in adolescent males with ADHD (n = 17), and also compared WM microstructure relative to controls (n = 17). Significant associations (*p* < 0.05, corrected) were found between fractional anisotropy/radial diffusivity and ASD trait severity (positive and negative correlations respectively), mostly in the right posterior limb of the internal capsule/corticospinal tract, right cerebellar peduncle and the midbrain. No case–control differences were found for the diffusion parameters investigated. This is the first report of a WM microstructural signature of autistic traits in ADHD. Thus, even in the absence of full disorder, ASD traits may index a distinctive underlying neurobiology in ADHD.

## Introduction

Attention-deficit/hyperactivity disorder (ADHD) is the most common childhood-onset neurodevelopmental disorder, with a male:female prevalence bias of 2–3:1 in general population samples (Polanczyk et al. 2007, Ramtekkar et al. 2010). It is associated with intellectual disability (ID) (Dykens 2000) and other neurodevelopmental conditions, notably autism spectrum disorder (ASD) (Rommelse et al. 2010). This comorbidity with ASD occurs at both trait (e.g. Ronald et al. 2008, 2010) and full disorder (e.g. Clark et al. 1999, Yoshida and Uchiyama 2004) level and contributes to clinical and developmental variability in ADHD (Cooper et al. 2013).

Standard structural and functional magnetic resonance imaging (MRI) has shown many brain abnormalities in ADHD (Cortese and Castellanos 2012). For example, volumetric decreases in lobar white (WM) have been found (e.g. McAlonan et al. 2007; Castellanos et al. 2002), and meta-analyses have identified localised volumetric grey matter (GM) abnormalities in the basal ganglia (Nakao et al. 2011; Frodl and Skokauskas 2012). Dysfunction has been identified in a variety of networks, including those related to executive function and attention (Cortese et al. 2012). However, there is no diagnostic neurobiological marker for ADHD and it has become apparent that localised alterations in brain structure and function are unlikely to provide a unifying explanation for its complex and heterogeneous clinical presentation (Cherkasova and Hechtman 2009; Konrad and Eickhoff 2010). Diffusion MRI, including diffusion tensor imaging (DTI) (Basser et al. 1994; Basser and Pierpaoli 1996), uses the diffusion of water to infer properties of WM microstructure (Beaulieu 2002), providing another means of investigating neural circuitry in ADHD. The most investigated diffusion parameters are fractional anisotropy (FA, the proportion of the tensor that can be ascribed to anisotropic diffusion) and mean diffusivity (MD, the orientationally averaged apparent diffusion coefficient within each voxel) (Basser and Pierpaoli 1996). Axial and radial diffusivities (AD and RD, diffusivity parallel and perpendicular to axons respectively) can also be investigated.

The relationship between phenotypic variability in ADHD and alterations in WM microstructure is not yet clear. In ADHD case–control studies, various analytical methods (whole brain and region-of-interest approaches, and voxel-based and tract-based techniques) have been used to investigate correlation between clinical indices of phenotypic variability and diffusion metrics. Results have varied in terms of the measure used and the anatomical location where correlations were found. Whilst some studies have found associations between phenotypic measures and white matter microstructure (e.g. Casey et al. 2007; Nagel et al. 2011; Konrad et al. 2010, 2012; Chuang et al. 2013; Shang et al. 2013) others have not (e.g. Hamilton et al. 2008; Dramsdahl et al. 2012; Silk et al. 2009a, b). In a large sample of typically developing young children, correlations have been found between measures of inattention and hyperactivity/impulsivity and FA (Qiu et al. 2012).

No studies have examined the association between indices of tissue microstructure and autistic traits in ADHD. However, the high clinical comorbidity between these two neurodevelopmental conditions and increasing evidence for their genetic overlap [reviewed in Rommelse et al. (2010)], which are reflected in the decision to allow their co-diagnosis in one individual in DSM-5, makes the study of this association highly relevant. Spatially distributed alterations in WM microstructure in ASD [reviewed in Travers et al. (2012)], which overlap with those reported as abnormal in ADHD (see below), provide additional suggestion of shared neurobiology. The most consistent locations of ASD case–control differences (Travers et al. 2012) have been found in the corpus callosum (e.g. Barnea-Goraly et al. 2004; Shukla et al. 2011; Jou et al. 2011), cingulum (e.g. Kumar et al. 2010; Shukla et al. 2011; Weinstein et al. 2011) and temporal lobe (e.g. Ke et al. 2009; Barnea-Goraly et al. 2010; Noriuchi et al. 2010). ASD traits in ADHD have also been found to index phenotypic severity in terms of clinical and cognitive deficits, independently of the extent of ADHD symptomatology (Cooper et al. 2013), further suggesting autistic traits may provide an index of brain abnormalities in ADHD.

The heterogeneity in the association of phenotypic measures with diffusion indices is mirrored by variable results in ADHD DTI case–control comparisons. Since the first of these studies (Ashtari et al. 2005), distributed differences in diffusion parameters have been reported, again using diverse analytical techniques. Where differences have been described in specific WM pathways as opposed to in terms of broader brain regions, these have included the corpus callosum (e.g. Cao et al. 2010; Dramsdahl et al. 2012), cingulum (e.g. Konrad et al. 2010; Makris et al. 2008), frontostriatal tracts (e.g. de Zeeuw et al. 2012a), corticospinal tract (e.g. Hamilton et al. 2008), superior longitudinal fasciculus (e.g. Hamilton et al. 2008; Makris et al. 2008), inferior longitudinal fasciculus and inferior fronto-occipital fasciculus (e.g. Konrad and Eickhoff 2010), anterior thalamic radiation (e.g. Konrad et al. 2010) and other thalamic connections (e.g. Xia et al. 2012), anterior corona radiata (e.g. Kobel et al. 2010) and the internal capsule (e.g. Pavuluri et al. 2009). Several of these studies in fact find many widely distributed differences, and there are reports of bidirectional changes in diffusion parameters in different areas. Whilst many observed differences are of exclusively decreased FA relative to controls (e.g. Hamilton et al. 2008; Makris et al. 2008; de Zeeuw et al. 2012a; Xia et al. 2012) increases have also been reported (e.g. Davenport et al. 2010; Li et al. 2010; Kobel et al. 2010). Where other parameters have been examined, increases in MD are usually (although not exclusively) found, but do not necessarily co-localise with regions of FA change. Two studies report no case–control differences in diffusion parameters (Silk et al. 2009a; de Zeeuw et al. 2012b).

A few recent ADHD studies have used tract-based spatial statistics (TBSS) (Smith et al. 2006), which allows whole-brain WM analysis whilst decreasing the impact of imperfect spatial normalization and reducing the number of independent comparisons relative to other voxelwise methods. ADHD TBSS studies in young people have found widely distributed case–control differences in diffusion indices in middle childhood (Nagel et al. 2011), throughout middle childhood and adolescence (Silk et al. 2009b) and during later adolescence (Chuang et al. 2013, Tamm et al. 2012). Such differences have also been found in adults whose ADHD had been diagnosed in childhood (Cortese et al. 2013).

Drawing together the results of studies to date is challenging, but a meta-analysis of voxelwise ADHD case–control diffusion studies has found that reported changes in diffusion parameters are widespread, with the most reliable alterations (either decreased or increased FA) located in the right anterior corona radiata, right forceps minor, bilateral internal capsule and left cerebellum (van Ewijk et al. 2012). In addition to the heterogeneity of analytical methods, comparison and synthesis of diffusion findings is complex because of disparity in the types of clinical cases included across studies. An important source of variability in ADHD arises due to comorbidity. The primary aim of this paper is thus to focus on associations between comorbid ASD traits and diffusion parameters in male adolescents with ADHD.

## Methods

### Recruitment and Clinical Variables

Forty male participants (19 ADHD, 21 controls) were recruited. All were required to be right-handed as handedness and gender have independent associations with diffusion indices (e.g. Westerhausen et al. 2004).

Individuals were aged 14 years 0 months–18 years 11 months at the point of scanning, and had IQ test scores of >70 assessed using WISC-IV (Wechsler 2004). All subjects were Caucasian back to their grandparents. Ineligibility was conferred by epilepsy, significant head injury, psychosis, ASD, contraindication to MRI scanning, or any other known medical condition (including extreme prematurity or very low birth weight) with potential to affect brain development. With the exception of ADHD medication in the patient group, subjects were otherwise psychotropic medication-naïve. To minimise motion, subjects taking ADHD medication at the point of the scan were not required to stop it. Written informed consent was obtained from parents and young people aged 16–18; those aged 14–15 gave assent. Approval was gained from South East Wales NHS Research Ethics Committee.

The ADHD group was a subset of participants who had previously taken part in a study of genetic and environmental influences on ADHD (described in Stergiakouli et al. 2012). All cases had a lifetime diagnosis of DSM-IV ADHD-combined type (ADHD-C) that had been confirmed by the parent version of the Child and Adolescent Psychiatric Assessment (CAPA) (Angold et al. 1995), a research diagnostic interview. Information from teachers had confirmed pervasiveness. The control group were volunteers who responded to study advertisements in public places.

### Additional Clinical Data on Cases and Controls

Parent-rated questionnaires were used to assess demographics and provide additional trait measures of psychopathology. A modified version of the DuPaul ADHD scale (DuPaul 1981) measured current ADHD severity. Nine items correspond to DSM-IV hyperactive-impulsive symptoms and nine to inattentive. Each item was endorsed as present ‘not at all’, ‘just a little’, ‘pretty much’, or ‘very much’, with corresponding scores for each endorsement coded as 0–3. From this, a total ADHD symptom score of 0–54 and subscale scores for hyperactivity/impulsivity and inattention (both 0–27) were calculated. The Social Communication Questionnaire (SCQ, previously known as the Autism Screening Questionnaire, ASQ) (Berument et al. 1999), a 40-item questionnaire based on the Autism Diagnostic Interview-Revised (ADI-R) (Lord et al. 1994), was used to assess autistic traits and give a trait score from 0 to 40. Total SCQ scores were further divided into sub-domains of social deficits (0–20), communication deficits (0–10) and repetitive behaviours (0–8) according to DSM-IV ASD diagnostic symptoms (described further in Cooper et al. 2013). Global burden of current psychopathology was assessed using the Strengths and Difficulties Questionnaire (SDQ) (Goodman 1997).

### Scanning Procedure

Participants were acclimatised to the process using a ‘mock’ scanner just prior to the scan itself. Subjects underwent the scan in a 3T GE (General Electric) HDx MR system. An 8 channel head coil was used. The ASSET factor was 2. TE was 87 ms and TR was 15–20 R–R intervals (i.e. 13.8–18.5 s assuming an average heart rate of 65 beats per minute). Data were acquired with a field of view of 230 × 230 mm with an acquisition matrix of 96 × 96 (subsequently zero-filled to 128 × 128 prior to the Fourier transform). Diffusion-weighted data were acquired with a twice-refocused spin-echo echo-planar imaging sequence giving whole oblique axial brain coverage, with 60 slices of 2.4 mm thickness aligned parallel to the anterior commissure-posterior commissure line. The b value was 1200 s/mm^2^. Diffusion data were acquired with diffusion encoded along 60 non-collinear optimally ordered directions (Jones et al. 1999; Cook et al. 2007), with six non-diffusion-weighted scans initially. Data were acquired using peripheral gating to the cardiac cycle, in all but one subject in whom this was not possible due to tachycardia. Diffusion imaging acquisition time was around 20–28 min depending on the subject’s pulse rate.

### Data Processing

All diffusion data processing was performed using ExploreDTI, version 4.8.2 (Leemans et al. 2009). Images were corrected for subject motion and eddy current distortion (Haselgrove and Moore 1996) with appropriate reorientation of the encoding vectors to account for subject rotation (Leemans and Jones 2009). A tensor model was then fitted to each voxel in the data using non-iterative weighted linear least squares regression. Residuals to the tensor fit were examined to look for data points that were outliers, and subjects whose scans showed significant artefact were excluded from all analyses (n = 2 from each group, resulting in 17 young people with ADHD and 19 controls with usable diffusion data). The tensor was then re-estimated using the Robust Estimation of the Tensor by Outlier Rejection (RESTORE) algorithm (Chang et al. 2005). RESTORE endeavours to correct for data artefacts due to physiological noise, such as subject motion and cardiac pulsation. It uses a stringent regression model to exclude outliers and then re-computes the tensor with higher accuracy. From the tensor model estimated with RESTORE, the quantitative metrics of FA, MD, AD and RD were estimated. If significant results were seen for FA or MD for either correlation or case–control analyses, these were followed up by examining AD and RD to allow further inference of the potential origin of changes.

### Tract-Based Spatial Statistics (TBSS)

Voxelwise statistical analysis of FA, MD, AD and RD data was carried out using TBSS (Smith et al. 2006), part of FMRIB Software Library (FSL) (Smith et al. 2004). TBSS projects all subjects’ FA data onto a mean FA tract skeleton, before applying voxelwise cross-subject statistics. The skeleton was thresholded at FA ≥ 0.3. The output used threshold-free cluster enhancement-based cluster inference (Smith and Nichols 2009).

### Data Analyses

Mann–Whitney U tests were used to analyse group differences in the distribution of demographic and clinical variables.

General linear models were generated to test for correlations between diffusion parameters and ASD/ADHD trait measures in the ADHD group. Where significant correlations were found for total trait scores, the relative contribution of sub-domain scores were investigated post hoc. Further models were generated for the following analyses: case–control differences in diffusion parameters, the impact of age on case–control differences in diffusion parameters and the correlation of diffusion parameters with age across the total sample, and the effect of age on diffusion indices according to group. Statistical significance for TBSS results was considered as *p* < 0.05 after correction for multiple comparisons. For visualisation/presentation of TBSS results, voxels showing significant values were enhanced using the tbss_fill function in FSL.

IQ was not included as a covariate as it is systematically and inextricably related to the defining cognitive-behavioural characteristics of ADHD, thus it is impossible to statistically control for the variance associated with it without removing meaningful variance in the key measures of interest (Miller and Chapman 2001; Dennis et al. 2009).

## Results

### Participant Inclusion in Analyses

The correlation analyses in the ADHD group were run with all 17 ADHD participants who had usable diffusion data. Two controls were excluded from the case–control analyses due to high endorsement of hyperactive-impulsive/inattentive symptoms, leaving 17 young people with ADHD and 17 controls in the main case–control analyses. However, two subjects in the ADHD group showed very low current ADHD symptom levels. They were considered ‘remitted’ and all case–control analyses were subsequently re-run excluding them (i.e., with 15 ADHD and 17 controls), to ensure that their inclusion was not altering results.

Participant characteristics of all those in the main analyses (17 ADHD, 17 controls) are shown in Table 1. All control subjects were in the normal range for total difficulties score on the SDQ and none had any SDQ subscale score in the abnormal range. The majority (16/17) of the ADHD group had a history of treatment with short/long acting stimulants and/or atomoxetine. Six had had their medication discontinued for clinical reasons. Of the 15 cases with persistent ADHD, 14/15 had a history of medication treatment.

**Table 1:** Demographic and clinical sample characteristics

	ADHD group (n = 17)		Control group (n = 17)	
	Mean	SD, range	Mean	SD, range
Age, years	15.6	(1.3, 14.3–18.6)	16.9	(1.2, 15.0–18.8)
Full scale IQ	87.6	(9.8, 75–110)	106.9	(7.6, 98–122)
DSM-IV HI score (max 27)	18.7	(7.3, 2–26)	1.94	(2.2, 0–7)
Mean DSM-IV I score (max 27)	19.1	(5.0, 6–27)	2.6	(2.9, 0–8)
Mean total ADHD symptoms score (max 54)	37.8	(11.5, 8–53)	4.5	(4.2, 0–14)
Mean total SCQ score (max 40)	7.1	(5.9, 1–22)	1.0	(1.1, 0–3)
Mean SDQ difficulties score (max 40)	19.1	(8.0, 5–35)	2.9	(2.5, 0–8)
Family income group^a^				
Low (%)	50		5.9	
Medium (%)	50		11.8	
High (%)	0		82.4	
Took medication on morning of scan day				
Yes	10		N/a	
No	6		N/a	
Unknown	1		N/a	

*HI* hyperactive-inattentive, *I* inattentive, *SCQ* Social Communication Questionnaire, *SDQ* Strengths and Difficulties Questionnaire

^a^Low: annual household income <£20,000, medium: annual household income £20–40,000, high: annual household income >£40,000 (information not available for one participant with ADHD)

There were significant differences between the 17 ADHD cases and 17 controls in the distributions of family income status (U = 252, *p* < 0.001) and full scale IQ (U = 265, *p* < 0.001). There was a significant difference in the distributions of age (U = 223, *p* = 0.006), with the ADHD group being slightly younger (difference in mean age = 1.3 years). There were also group differences in DSM-IV ADHD symptom scores (hyperactive-impulsive, inattentive and total score; U = 8, 3.5 and 4 respectively, all *p* < 0.001) and SCQ score (U = 19, *p* < 0.001). Within both the ADHD and control groups, age was not correlated with either total ADHD or SCQ scores.

### Association with Level of ASD Symptoms and ADHD Severity

**Fig. 1:**
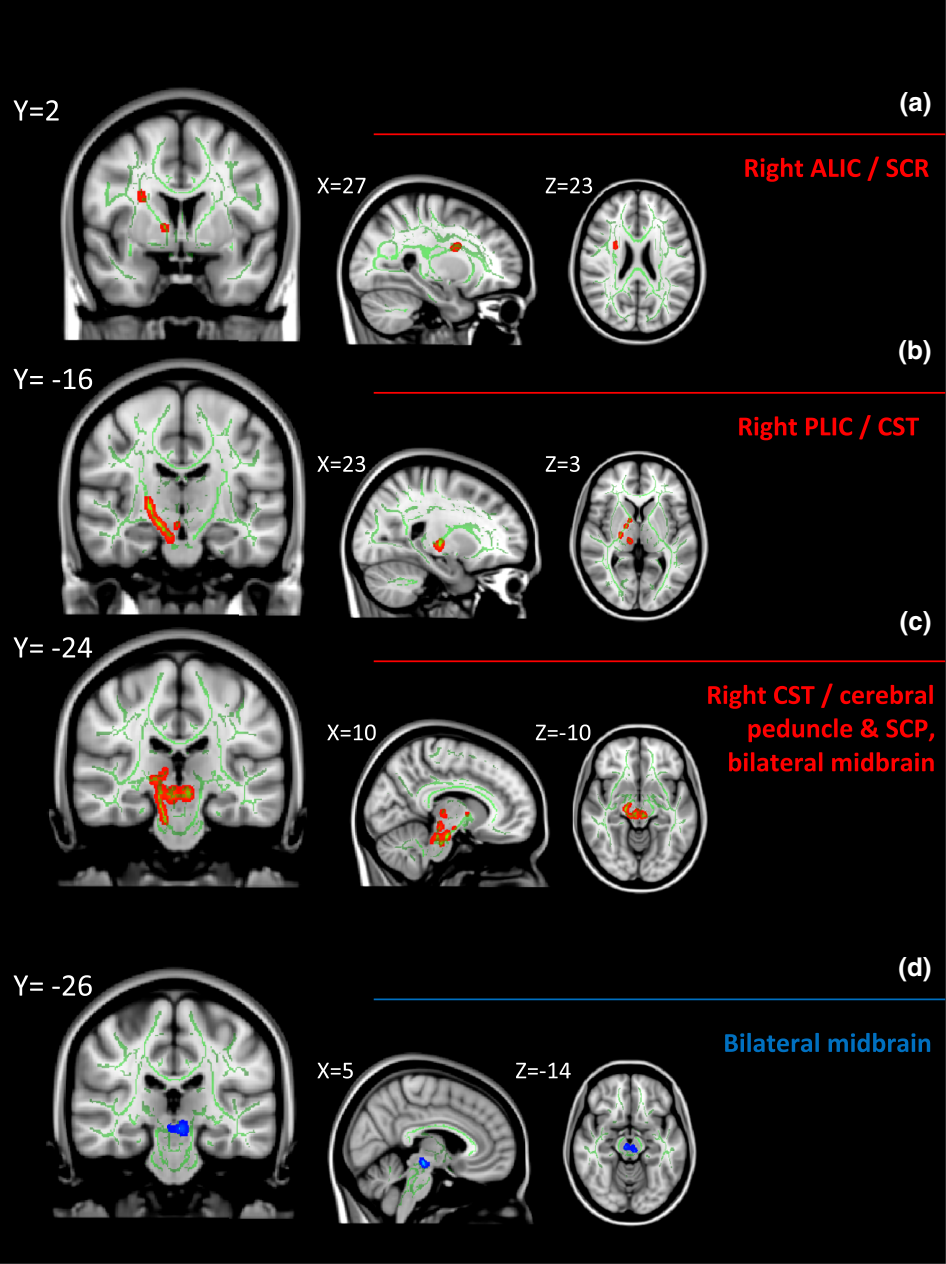
Areas of correlation between diffusion parameters and SCQ score. *a*–*c* Areas of significant (*p* < 0.05, corrected) positive correlation between FA and SCQ score (*red*). *d* Areas of significant (*p* < 0.05, corrected) negative correlation between RD and SCQ score (*blue*). *SCQ* social communication questionnaire, *FA* fractional anisotropy, *RD* radial diffusivity, *ALIC* anterior limb of the internal capsule, *SCR* superior corona radiata, *PLIC* posterior limb of the internal capsule, *CST* corticospinal tract, *SCP* superior cerebellar peduncle. Results overlaid on the MNI152 T1 1 mm brain. The mean FA skeleton is shown in *green*. Voxels showing significant values are enhanced using the tbss_fill function in FSL (Color figure online)

In the ADHD group (n = 17), significant positive correlation was found between FA and autistic traits as indexed by SCQ score (Fig. 1). The most extensive areas of correlation were seen in the inferior section of the right posterior limb of the internal capsule, including the corticospinal tract down into the crus cerebri of the cerebral peduncle, and also in the right superior cerebellar peduncle, right medial lemniscus and the midbrain bilaterally. Smaller areas of correlation were seen in the right anterior limb of the internal capsule and superior corona radiata. Significant negative correlation was also found between RD and SCQ score in the midbrain bilaterally. There were no correlations with MD or AD. No correlation was observed between FA or MD and ADHD severity as indexed by total ADHD score.

**Fig. 2:**
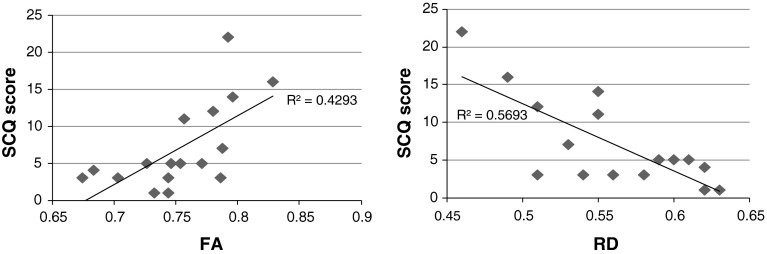
Association between FA/RD and SCQ score at representative voxels where correlations significant at *p* < 0.05, corrected. *SCQ* social communication questionnaire, *FA* fractional anisotropy, *RD* radial diffusivity. Units for radial diffusivity = 10^−3^ mm^2^ s^−1^. Linear trendlines are shown. The FA plot shows correlations at a voxel in the right posterior limb of the internal capsule/corticospinal tract at the level of the cerebral peduncle, significance at this voxel is *p* = 0.038, corrected. The RD plot shows correlations at a voxel from the midbrain just left of the mid-sagittal plane, significance at this voxel is *p* = 0.042, corrected

Representative voxels from the skeleton were examined with respect to the nature of the correlation between FA/RD and SCQ score (Fig. 2).

Given the observed correlations between FA/RD and total SCQ scores in the ADHD group, the relative contributions of SCQ sub-domain scores were examined post hoc. As social and communication sub-domain scores were highly cross-correlated (Spearman’s rho 0.660, *p* < 0.001), correlations of diffusion parameters with summed socio-communicative scores and repetitive behaviour scores were tested separately. A similar pattern of results was seen as for total ASD score, but with associations being driven by socio-communicative scores with no contribution from repetitive behaviour scores. RD remained significantly negatively correlated with socio-communicative scores (*p* < 0.05), although correlations between FA and socio-communicative scores no longer quite reached significance (0.05 < *p* < 0.08).

### Case–Control Analyses

No significant group differences in FA or MD were found in the initial analysis (n = 17 cases, 17 controls). The addition of age as a covariate did not unmask any group differences and diffusion parameters showed no correlation with age across the total sample. Also, no age × group interactions on diffusion parameters were found. Results were unchanged when the two ‘remitted’ ADHD participants were excluded (i.e. analysing n = 15 cases and 17 controls).

The addition of IQ as a covariate into the initial case–control analysis (n = 17 cases, 17 controls) did not alter results.

## Discussion

### Association with Level of ASD Symptoms and ADHD Severity

The correlation analysis of the association of ASD and ADHD traits with diffusion parameters in the ADHD group showed several areas where autistic traits were associated with altered tissue microstructure. When total SCQ score was decomposed into socio-communicative scores and repetitive behaviours, correlations seemed to be driven by the former, although they fell below significance for FA. However, variability in sub-domain scores will have been restricted because those with an ASD diagnosis were not included.

The most extensive areas of (positive) correlation with autistic symptoms were seen for FA in the right posterior limb of the internal capsule including the corticospinal tract. This is an area where ADHD case–control differences have been reported (e.g. Hamilton et al. 2008, reduced FA in the corticospinal tract; Nagel et al. 2011, reduced MD in the posterior limb of the internal capsule). Although this is not an area with extensive reports of ASD case–control differences (ASD at diagnostic levels), *decreases* in FA in this area have been reported (Brito et al. 2009; Shukla et al. 2011); though associations between autistic behavioural measures and diffusion parameters in the general population have not been widely investigated and have never been examined in those with ADHD. However, there is some support for potential involvement of the corticospinal tract in social cognition, as well as its known role in motor function, from ASD case–control studies using transcranial magnetic stimulation (Oberman et al. 2012; Enticott et al. 2012). It is of note TBSS does not provide anatomical specificity for individual fibre bundles though, and thus the potential involvement of other fibre systems passing through the posterior limb of the internal capsule, for example corticobulbar fibres and the superior thalamic radiation, cannot be ruled out.

Positive correlation between socio-communicative score and FA was also seen in the right superior cerebellar peduncle in the ADHD group. Abnormal diffusion parameters in the cerebellar area have been reported in ASD case–control studies (e.g. Sivaswamy et al. 2010; Catani et al. 2008; Cheung et al. 2009). Correlations were examined in the latter two studies, with *negative* correlations found between FA and ADI-R social scores in Catani et al. (2008) and between FA and ADI-R repetitive behaviour scores in Cheung et al. (2009). A further area of positive correlation between autistic symptoms and FA, with associated negative correlation with RD, was seen in the midbrain in the present study; as were smaller areas of (positive) correlation with FA only in the right anterior limb of the internal capsule and superior corona radiata. Again, these are not areas with extensive report of ASD case–control differences, although reduced MD has been noted in the brainstem in Asperger’s syndrome (Bloemen et al. 2010), and decreases in FA have been noted in the anterior limb of the internal capsule (Shukla et al. 2011a, b).

The differences in the presence and direction of correlation of ASD traits with diffusion parameters found in the present study imply autistic traits may relate to brain microstructure differently in ADHD. As the relative contributions to diffusion indices from axon density, diameter and myelination cannot be inferred from the tensor model, it is challenging to interpret the meaning of AD and RD and extreme caution should be taken when interpreting changes in RD as proxy markers for myelination (Wheeler-Kingshott and Cercignani 2009). However it is at least possible to speculate that the observed increase in ASD symptomatology may reflect either more dense packing of fibres, decreased neural branching or increased myelination; or a combination of these factors. Nonetheless, it may be an oversimplification to assume that decreased FA may universally reflect increasing severity in ADHD and autistic symptomatology throughout the whole brain, across the lifespan. Variations in diffusion parameters can index several aspects of microstructural anatomy (Beaulieu 2002) and higher FA may not necessarily represent advantage in terms of its cognitive or behavioural correlates (Thomason and Thompson 2011). No associations were found with current ADHD severity; it is however possible that autistic traits in ADHD may represent more enduring and stable markers of impairment than do ADHD traits themselves. Further evidence for the biological overlap of the two conditions is provided by recent analyses of functional MRI data—a sustained attention task (Christakou et al. 2013) and a graph theoretical analysis of resting state data (Di Martino et al. 2013), which find shared and discrete abnormalities of function and connectivity in ASD and ADHD.

No associations were found between current levels of ADHD traits and diffusion parameters. However, ADHD symptoms are dynamic (Willcutt et al. 2012), thus by adolescence the relationship between current ADHD severity and WM microstructure is likely to be complicated by, for instance, the preceding trajectory of symptoms (progression or attenuation) and response to medication.

### Case–Control Analyses

In this sample of adolescents with ADHD, no case–control differences in any diffusion parameters were found. Reported findings from previously published TBSS studies in young people with ADHD are mixed in terms of the location and direction of case–control diffusion differences found (Nagel et al. 2011; Chuang et al. 2013; Silk et al. 2009b; Tamm et al. 2012), as they are in the wider DTI ADHD literature, where null results are also reported (Silk et al. 2009a; de Zeeuw et al. 2012b). However, notably, only the minority of case–control studies assess a predominantly older adolescent age range (Chuang et al. 2013; Tamm et al. 2012). It is complex to interpret overall findings from studies where a broad age range is included and to extrapolate findings across studies with different age ranges. This is due to the influence of age on WM (e.g. Lebel et al. 2008) and suggestion that the ADHD brain may have an abnormal maturational trajectory (Shaw et al. 2007). Although the present groups had a statistically significant difference in age distribution, the magnitude of the difference was small and the age range was relatively narrow, which may have precluded age exerting an effect as a covariate. Whilst no age x group interactions on diffusion parameters were found, this does not preclude preceding differences in the developmental trajectory of WM, which cannot be inferred without longitudinal data.

Medication may have helped normalise changes seen in other studies. The majority of the ADHD group inevitably have varying duration of stimulant or atomoxetine treatment. However, in such a sample of adolescents it would be unusual to find many without preceding pharmacological intervention—a small UK study found 91 % of those diagnosed with ADHD are prescribed medication (Parr et al. 2003). The underlying mechanism by which medication may contribute to normalising developmental brain changes is not understood. However there is evidence that stimulants may normalise both brain function (e.g. Rubia et al. 2009) and aspects of WM (Castellanos et al. 2002) and GM (Nakao et al. 2011) macrostructure, including the trajectory of cortical development (Shaw et al. 2009). Many ADHD diffusion studies have included pharmacologically-treated participants although they have varied in the requirement for a washout/withholding period. Some DTI studies where the ADHD group has had mixed medication status have explored the impact of stimulants but, conversely, have not found medication status to alter results (Ashtari et al. 2005; Hamilton et al. 2008; de Zeeuw et al. 2012a) although such analyses have resulted in small subgroups. In the current analysis, our sample size did not permit a stratified analysis. Whether stimulants affect diffusion parameters in the immediate as opposed to in the longer term is not yet known. However, as no functional scans were carried out as part of the present study, it was decided that subjects would not be required to stop medication in order to improve scan quality.

However, more caution may potentially be needed in the interpretation of group differences in previous studies for the following reasons. (1) Imperfect matching at a group level on influential confounding variables such as handedness and gender could generate systematic differences between groups which cannot meaningfully be covaried out at the analysis stage. (2) ADHD, surplus to its core features, is associated with multiple, sometimes subtle, potentially dynamic cognitive, behavioural and developmental problems and it may be that these features are underpinned by altered WM development. (3) Withholding medication could predispose to movement during scanning, as could including younger children. Without rigorous compensation in processing, group level discrepancies in movement could generate spurious differences. There is evidence that distributions of certain case–control differences in WM microstructure in autism could be artefactual with the authors advising caution in interpretation of such findings (Walker et al. 2012). (4) Traditional voxel-based morphometry-style analyses of DTI data are constrained by an arbitrary choice of smoothing kernel size because it is unlikely that the effect size of any potential group differences in neurodevelopmental/psychiatric disorders will be known in advance. However, choosing different sized Gaussian kernels for smoothing can produce highly discrepant locations of group FA differences (Jones et al. 2005). Of note, in the schizophrenia versus controls dataset analysed by Jones et al. (2005), no case–control differences were elicited with kernels less than 7 mm. (5) Finally, reporting of results that have not been corrected for multiple comparisons is an issue especially pertinent to whole-brain analyses, which require more stringent correction. Factors with potential to confound interpretation of results in the ADHD DTI literature are reviewed in detail in van Ewijk et al. (2012).

### Strengths and Limitations

All subjects were right-handed males to increase homogeneity on confounding variables. The groups were not IQ matched, although no young people met criteria for ID. However the mean IQ in the ADHD group is in keeping with a meta-analysis suggesting the effect size for the lowering in cognitive ability associated with ADHD equates to about a 9 point difference in full scale IQ (Frazier et al. 2004). Thus, the present IQ distribution increases the pragmatism and generalizability of results. This IQ difference might be expected to exacerbate case–control differences in typical development, but there is evidence that the expected relationship between IQ and neuroanatomy may be altered in ADHD (de Zeeuw et al. 2012b). Allowing medication to be continued where possible will have helped to homogenise the degree of motion during the scanning, between cases and controls. Further strengths are the use of high angular resolution diffusion imaging using 60 direction DTI with cardiac gating, plus use of the optimal regression strategy of the RESTORE algorithm, to ensure accurate tensor estimation. The majority of ADHD DTI studies use between 6 and 32 direction DTI, and only a couple use robust estimation routines such as RESTORE (de Zeeuw et al. 2012a, b; Peterson et al. 2011).

There was a small but statistically significant difference in age distribution, with the ADHD group having a mean age of 1.3 years lower than the controls. However, as this difference was small and there was no impact of age as a covariate, this suggests that it was not of consequence to the results. The groups also had differences in their socio-demographic status. However, it is challenging to recruit control volunteers from the less advantaged backgrounds (e.g. lower income, lower IQ) that typify clinic cases in the UK where health care is provided free of charge.

Whilst current ADHD trait measures were assessed, current subtype was not, although inclusion of only those with a rigorously assessed lifetime diagnosis of ADHD-C ensured relative sample homogeneity. However, it is known that levels of ADHD symptom dimensions vary longitudinally so individuals can switch between subtypes (Willcutt et al. 2012). Hence a dimensional approach to assessing current trait severity may actually be more practical. In line with DSM-IV convention at the time of recruitment, those with a known comorbid ASD diagnosis were not included in the ADHD group. Whilst this will have reduced the variability in autistic trait scores, including those with full ASD would not have allowed the question of how sub-threshold autistic traits relate to brain microstructure in ADHD to be addressed.

As with other DTI ADHD studies, this research is cross-sectional so no inference can be made about the developmental trajectory of diffusion parameters or the direction of observed effects. Results cannot be generalised beyond this age group nor extrapolated to females or left-handers.

The mean FA tract skeleton was generated from alignment of FA images to the FMRIB58_FA standard space image. This approach was taken because by later adolescence the brain should be nearing adult patterns of maturity. It would indeed be interesting to explore the impact of an adolescent-specific template in future larger studies.

Unless alterations in diffusion indices coincided with the voxels with the highest anisotropy (those from which the mean FA tract skeleton is generated), they would not be detected using TBSS. It may be that even with a comparable sample size to previous studies, this study did not have the power to detect effects given the multiple testing correction required. Although often reported as such, it is also not possible to infer a straightforward index of ‘connectivity’ from a voxel-averaged FA and diffusivity derived from the tensor, as the tensor model cannot resolve complex fibre architecture (Jones 2010; Jones et al. 2013). Hence the average FA or diffusivity within a voxel is not necessarily reflective of underlying tract morphology and true differences in the metrics of individual tracts may be missed. Tract-specific approaches (Jones et al. 2005), whereby diffusion information is integrated along the length of an anatomically defined WM pathway as opposed to considering differences at a voxel level, may have more sensitivity to detect alterations in microstructure (Kanaan et al. 2006; Keedwell et al. 2012). Lastly, as the relative contributions of individual elements of microstructure cannot be conclusively inferred from the tensor model alone, alternative methods are necessary to help resolve these considerations.

The present results should be interpreted as preliminary, requiring further investigation and replication in bigger samples, especially in view of the small sample size and it not being possible to fully match groups on age and family income. With regards to the case–control results, studies with smaller or comparable sample sizes have found group differences (e.g. Tamm et al. 2012, 12 ADHD, 12 controls; Kobel et al. 2010, 14 ADHD, 12 controls; Makris et al. 2008, 12 ADHD, 17 controls; Qiu et al. 2010, 15 ADHD, 15 controls; Peterson et al. 2011, 16 ADHD, 16 controls; Nagel et al. 2011, 20 ADHD, 16 controls), as have those with larger groups (e.g. Cao et al. 2010, 28 ADHD, 27 controls; Konrad et al. 2010, 2011, 37 ADHD, 34 controls; Dramsdahl et al. 2012, 29 ADHD, 37 controls; Cortese et al. 2013, 51 ADHD, 66 controls). These studies vary in the extent to which they match their groups on potentially influential confounding variables.

## Conclusions

This study is the first to report significant associations between tissue microstructure and autistic traits in ADHD and highlights the need for further investigation of the biological overlap of ADHD and ASD. Abnormal socio-communicative traits in ADHD appear to have association with the neurobiology of phenotypic variation in ADHD even in those who do not have a known ASD diagnosis. This highlights the need for clinicians assessing young people with ADHD to be vigilant to the potential impact of even sub-threshold socio-communicative difficulties.

Whilst no case–control differences in young people with ADHD were found in this analysis, in view of the previous abnormalities found by standard structural, functional and diffusion imaging, findings from this analysis should not be taken as an assertion that the brain does not display abnormalities in ADHD. Rather, TBSS may not have had the sensitivity in this sample to detect residual case–control differences which may have been rendered less pronounced by medication and developmental effects. Also, more caution may be needed in the interpretation of the previously reported widespread changes in diffusion parameters, particularly with regard to heterogeneity of samples across studies and the multiple comparisons issue.
